# The Corrosion Features of Q235B Steel under Immersion Test and Electrochemical Measurements in Desulfurization Solution

**DOI:** 10.3390/ma13173783

**Published:** 2020-08-27

**Authors:** Peng Gong, Guangxu Zhang, Jian Chen

**Affiliations:** School of Chemistry, Chemical Engineering and Life Science, Wuhan University of Technology, Wuhan 430070, China; gpeng2018@163.com (P.G.); Zhanggx2002@163.com (G.Z.)

**Keywords:** Q235B steel, desulfurization solution, corrosion mechanism, pitting corrosion

## Abstract

With the continuous tightening marine diesel engines emission standards, removing sulfur oxides (SO_X_) by sodium hydroxide solution absorption is a highly efficiency and economic method, which has been a hot area of research. The ensuing desulfurization solution is a new corrosive system, the aim of this paper is to ascertain the corrosion feature of Q235B steel in desulfurization solution, which lays a theoretical foundation for industrialization. For this purpose, mass loss, electrochemical techniques and surface analyses were applied. The results of mass loss highlight a reduction in the corrosion rate with 35 days of immersion. Higher exposure time increased the compactness of the corrosion product layer and changed phase composition. These conclusions are supported by surface analyses, such as X-ray diffraction and scanning electron microscope. However, electrochemical results showed that the polarization resistance *R*_p_ was fluctuant. Both of *R*_p_ and charge transfer resistance *R*_t_ reach a maximum after immersing 21 days. In addition, although the sediments attached to the steel surface could inhibit corrosion, pitting corrosion aggravated by hydrolyzation of FeSO_4_ should be given more attention.

## 1. Introduction

As human beings taking the environment into consideration severely, the effective disposal of sulfur oxides (SO_X_) in off-gas from marine diesel engine has been a worldwide puzzle and hot spots of research. Moreover, international Maritime Organization (IMO) had announced a stricter emission standard for SO_X_ in the regulation 14 [1]. In order to minimize the costs of tail gas up-to-standard discharge, it is an alternative method of reducing SO_X_ emissions by sodium hydroxide solution absorption [2]. Further, the technique has many advantages, such as high desulfurization efficiency (≥98%), no secondary pollution, sodium sulfate by-product as an industrial chemical, etc. At present, the chemical absorption mechanism, transfer mechanism and theoretical calculation approaches have been explored [3,4,5]. The desulfurization solution is a new corrosion system, therefore, the erosion problems of steel in desulfurization solution need to be investigated systematically for industrialization application early.

Ordinary carbon steels typical such as Q235B steels are being used as one of the main materials in China due to the shortage of resources and the consideration of economy. Therefore, it should give preference to Q235B steel in sodium hydroxide desulfurization system. However, it tends to rust when exposed to wet air, saltwater and other corrosive substances [6]. Corrosion would result in uneven steel surface, decreased thickness and deteriorated mechanical properties, further leading to perforations of pipelines and equipment failure. Until now, many studies have issued the corrosion mechanism and impacting factors of Q235B steel in different corrosive mediators. Cheng et al. [7] investigated the corrosion behavior of Q235B carbon steel in sediment from crude oil and found that corrosion pits were initiated under the scale deposits. Yu et al. [8] researched the atmospheric corrosion of Q235 steel in Turpan, indicating that the corrosion rate was 20 g·m^−2^·a^−1^ and the corrosion products were composed of α-FeOOH, γ-FeOOH, Fe_3_O_4_, Fe(OH)_3_. Sulfate ions is the most common ionic forms in desulfurization solution. Liu et al. [9] and Boah et al. [10] obtained the consistent conclusion that sulfate ion was even more corrosive than chloride ion. Xu et al. [11] demonstrated that sodium sulfate was harmful to the stability of the passive film. Interestingly, the presence of a larger amount of sulfate ions even inhibited the nucleation of the pitting of the steel. Whereas tail gas composition is complex, it is not hard to fathom that desulfurization solution would contain large amounts of metals, non-metals ions and organic substance. Great important should therefore be attached to give more insights into the corrosion feature of Q235B steel in desulfurization solution. Considering the requirement of industrialized application, the main objective of this paper was to research the corrosion behavior of Q235B steel in the desulfurization solution by open circuit potential (OCP), electrochemical impedance spectroscopy (EIS), polarization curves, scanning electron microscopy (SEM), X-ray diffraction (XRD), and mass loss. In addition, the corrosion mechanism was also discussed.

## 2. Experiment

### 2.1. Desulfurization Solution Analysis

Desulfurization solution was collected from an outdoor tank in Shanghai. pH was measured by a pH meter (PHS-3C, Shanghai Inesa Scientific Instrument Co., Ltd., Shanghai, China). Afterwards, desulfurization solution stored in a polystyrene vessel was sent to the lab for testing and chemical component analyses as soon as possible, the whole process was consistent with GB/T 5750-2006. An inductively coupled plasma-optical emission spectrometer (Prodigy 7) and an ion chromatograph (ICS-6000) were used to analyze its composition, in accordance with GB/T 8538-2016.

### 2.2. Immersion Test

Fifteen specimens with dimensions of 20 mm × 20 mm × 2 mm, were prepared for the immersion test at an ambient temperature. Prior to test, the specimens were ground smooth with emery papers ranging from grades 600 to 3000 to be up to the mustard of the corrosion tests. The specimens were then degreased with ethanol and dried in cool air. The initial mass and surface area of each specimen were measured by an electronic balance (BS110S) and a slide caliper (TM004), severally. The specimens were immersed in desulfurization solution for 7, 14, 21, 28 and 35 days. The specimens were retrieved at a scheduled time and immersed in a mixed solution (500 mL deionized water +3.5 g hexamethylenetetramine +500 mL 36% hydrochloric acid) vigorously for 30 s, followed by rinsing with water, dried with ethanol, and then weighted. Triplicate samples were taken for the measurements of the final mass after pickling. The corrosion rate of the specimen was calculated as follows:

(1) $$V_{\mathrm{corr}}=\frac{\left(M_{0}-M_{1}\right)\times 3650}{\rho st}$$

where *M*_0_ is the initial mass of the specimen; *M*_1_ is the final mass of the specimen after pickling; *ρ* is the density of Q235B steel; *s* is the surface area; and *t* is the immersion time. The element composition of Q235B steel manufactured by Sougang Mine Co (Qianan, China) is shown in Table 1.

### 2.3. Electrochemical Measurements

The measurements of the open circuit potential (OCP), electrochemical impedance spectroscopy (EIS), and polarization curves were performed on an electrochemical workstation (CS350H, Wuhan Corrtest Instrument Co., Ltd., Wuhan, China) conducted with a classical three-electrode cell (250 mL) at ambient temperature. The counter electrode was platinum wire electrode, and the reference electrode was Hg/Hg_2_SO_4_ electrode (MSE) connected to the cell via a Luggin capillary, which was filled with saturated potassium sulfate solution and 2% pure agar, all potentials were referred to it. The working electrode was Q235B steel, embedded in tetrafluoroethylene with an exposed area of 0.196 cm^2^. The working electrode was disposed the same as immersion test samples before the test itself began in order to ensure reliability of figures.

The EIS measurement was carried out with a perturbation signal of 10 mV AC potential versus the OCP in a frequency range from 10^5^ Hz to 10^−2^ Hz. The expectant data of EIS were obtained with a stabilized OCP and fitted with a suitable circuit model by a fit software named Zview. The polarization curves measurement was taken by changing the electrode potential automatically at a range from −200 mV to 150 mV vs. OCP with scan rate of 0.167 mV·s^−1^. In addition, three parallel tests were carried out and the representative value was reported.

### 2.4. Morphologies and Component Analysis

The surface morphology of the samples was carried out using scanning electron microscopy (SEM, JSM-IT300, Japan Electronics Co. LTD, Tokyo, Japan). The phase composition of corrosion products was analyzed by X-ray diffraction (XRD), XRD was carried out using RU-200B (Rigaku Corporation, Tokyo, Japan), with Cu-target, a tube voltage of 40 kV, a tube current of 30 mA, scanning range from 5° to 70°, and scanning step size of 2°/min.

## 3. Results and Discussion

### 3.1. Composition of the Desulfurization Solution

The analysis result of the desulfurization solution was shown in Table 2. The sample was analyzed for component cations and anions, such as SO_4_^2−^, SO_3_^2−^, Na^+^, K^+^, Zn^2+^ and so on. They are clearly believed to influence the corrosion process of metal, usually bringing about serious damage of the pipeline and equipment [12]. SO_4_^2−^ ions and Na^+^ ions were the highest concentrations of anion and cation, respectively, at 15,300.3 mg L^−1^ and 2785.5 mg L^−1^. The desulfurization solution was a mildly acidic medium with pH 6.95 ± 0.08. Additionally, there were some organics and insoluble solids in desulfurization solution, and the organic phase (PAHs) also has an effect on corrosion but only slightly.

### 3.2. Corrosion Morphology

**Figure 1:**
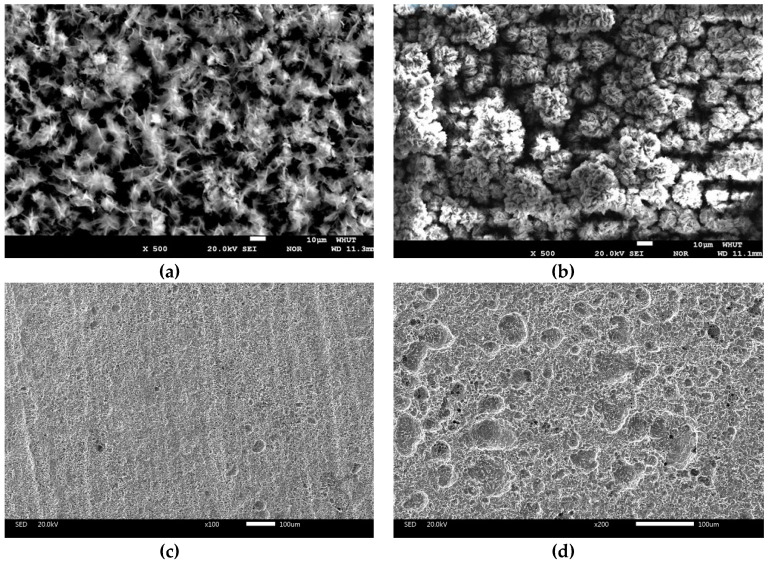
SEM macro-morphologies characteristics of Q235B steel in desulfurization solution with 7 days (**a**) and 35 days (**b**); The corresponding SEM macro-morphologies characteristics after the corrosion products were removed by acid pickling for 7 days (**c**) and 35 days (**d**).

After samples were immersed for 7 and 35 days, its morphologies showed obvious difference. The metallic luster of the samples surface was gradually lost and visible corrosion became more serious. As shown in Figure 1, for 7 days of immersion, general corrosion occurred on the steel surface, flocculent laurel-green precipitates were concatenated and scattered sporadically on the samples surface. After 35 days of immersion, the precipitates cover areas enlarged and thickened. The corresponding SEM photographs showed in Figure 1 after the rust was removed by acid pickling. It is obvious that the pit corrosion was slight in 7 days and subsequently performed increasingly serious with later 28 days of immersion.

### 3.3. Corrosion Products Analysis

**Figure 2:**
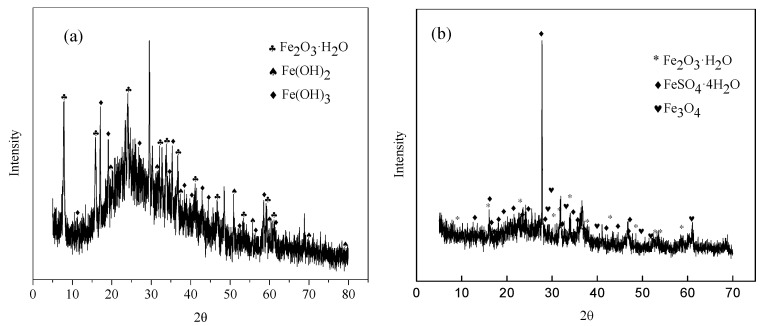
X-ray diffraction (XRD) analysis of corrosion products of Q235B steel in desulfurization solution with different immersion time (**a**) 7 days and (**b**) 35 days.

The XRD spectra of precipitates on the sample surface for 7 days and 35 days of immersion were shown in Figure 2. The results reveal that the primary corrosion products mainly consisted Fe(OH)_2_ (JCPDS3-903), Fe(OH)_3_ (JCPDS38-32) and Fe_2_O_3_·H_2_O (JCPDS13-92). After immersing for 35 days, corrosion film became thicker and more compact (Figure 1a,b), the end corrosion products were mainly composed of Fe_2_O_3_·H_2_O (JCPDS13-92), FeSO_4_·4H_2_O (JCPDS81-19) and Fe_3_O_4_ (JCPDS3-862). The phases changed enormously and are different from that of the steel in pure Na_2_SO_4_ solution [13].

### 3.4. Mass Loss and Corrosion Rate

**Figure 3:**
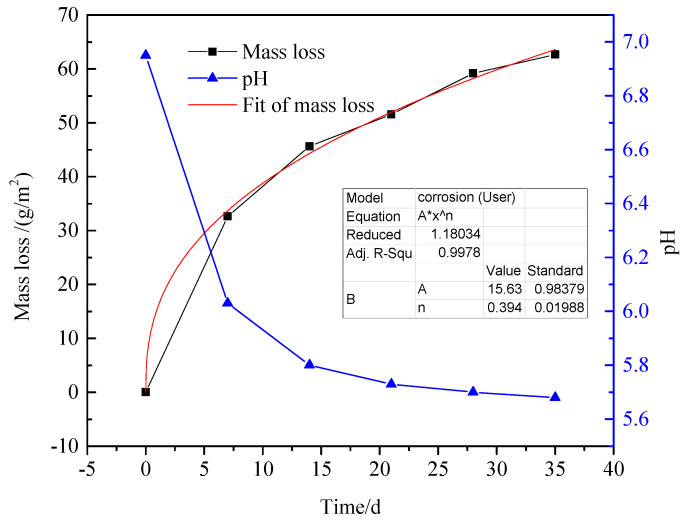
Curve of mass loss and pH with time of Q235B steel in desulfurization solution.

The relationship between mass loss and immersion time was presented in Figure 3. The mass loss of the samples increased continuously with prolonging the immersion time. It indicated that the samples were incessantly subjected to corrosion. Table 3 listed the time dependence of the corrosion rate (Δ*V*_corr_) reckoned from the mass loss for 35 days. The (Δ*V*_corr_) reduced sharply in the first 21 days of immersion. It had been reported that compact corrosion products film such as Fe_3_O_4_ on the matrix surface could form an effective anticorrosive film [14]. Therefore, the corrosive ion in desulfurization solution would not contact accessibly with the matrix and the (Δ*V*_corr_) decreased. However, corrosion pits were found on the matrix surface after the corrosion products were removed (Figure 1c,d). There were a few pitting holes dispersing on the surface after 7 days of immersion, while the local corrosion became severe with time. The variation of (Δ*V*_corr_) back up the conclusion. In the meantime, pH of the corrosion electrolyte decreased with time. It was positively associated with extent local corrosion and negatively correlated with mass loss. It could be inferred that local corrosion was dominant in the later stage of immersion, and there were corrosion reactions forming hydrogen ions.

### 3.5. Open Circuit Potential Measurements

**Figure 4:**
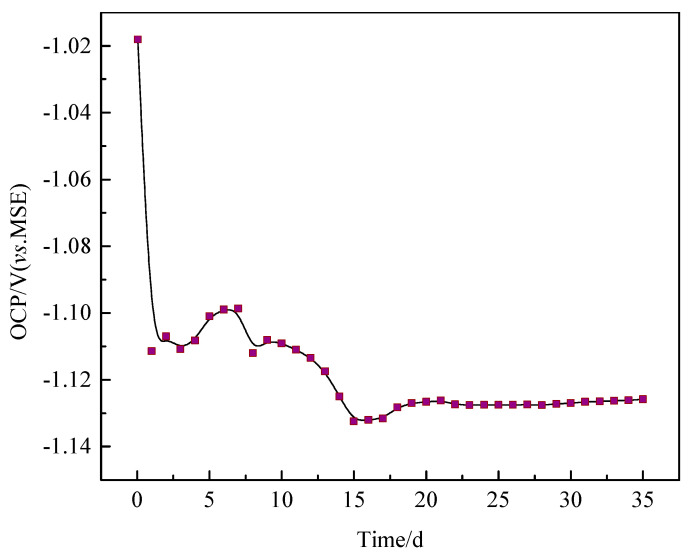
The trend of open circuit potential (OCP) for Q235B steel in desulfurization solution.

The relationships between OCP and immersion time of Q235B steel in desulfurization solution were shown in Figure 4. The OCP decreased sharply from –0.018 V to –1.111 V with time at the first day. Such a reduction was caused by an accelerated anodic reaction rate, according to mixed potential theory [15]. Three days later, the OCP augment a little and then renewed to reduce. The augment of the OCP attributed to the suppression of anodic reaction which might be induced by the accumulation of precipitates (Figure 1a,b) [16]. Whereas corrosion products began to form on the steel surface as the OCP continued to decline monotonically. It indicated that the gradually decreased of the OCP was caused by the adsorption for anion and an accelerated anodic reaction, due to the local corrosion (Figure 1c,d) [17]. Additionally, on subsequent days, the OCP reach a comparatively steady-state value (–1.127 V). This phenomenon manifested that cathodic reaction and anodic reaction achieved a balance [18].

### 3.6. Electrochemical Impedance Spectroscopy

**Figure 5:**
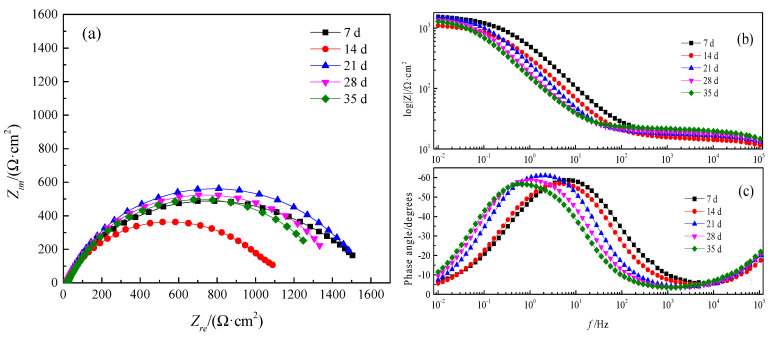
Electrochemical impedance spectra (EIS)of Q235B steel in desulfurization solution with 7 days, 14 days, 21 days, 28 days and 35 days, respectively; (**a**): Nyquist plot, (**b**): Bode magnitude, (**c**): Phase angle plot.

**Figure 6:**
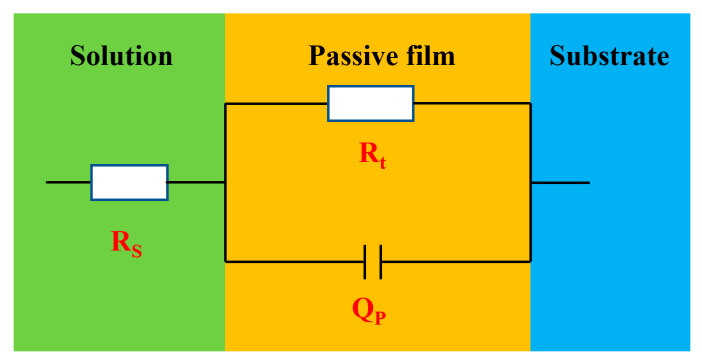
Equivalent circuits *R*_s_(*R*_t_*Q*_t_) used in the fitting procedure of the EIS experimental data.

The EIS was utilized to research interface reaction ability and the electrons migration in the corrosion products film [19]. The results of EIS measurements of Q235B steel at OCP in desulfurization solution with different immersion time were presented in Figure 5, containing Nyquist plots and Bode plots. The Nyquist spectrum (Figure 5a) indicated a single narrow capacitance loop for all specimens, which manifested that the electrode was not a pure capacitor [20,21]. It was likely related to the compactness and distribution of precipitates. Additionally, the diameter of the capacitive semicircle increased with time, which indicated the improvement of corrosion resistance. In Bode plots (Figure 5c), the maximum phase angle values approached about 60°, which demonstrated that the corrosion products film was porous [22]. Figure 5b revealed that there was only one time constant under the range of the frequency measured. Consequently, the one-time constant equivalent electrical circuits *R*_s_(*R*_t_*Q*_t_), as shown in Figure 6, is suitable to fit the experimental data. In the electrical analog circuits, *R*_s_ represents the resistance of solution, and *R*_t_ corresponds to the charge transfer resistance, linked to the resistivity properties of the passive film. Moreover, *Q*_t_ is a constant phase element (CPE) related to the dispersion of a double layer capacitance of the corrosion product layer [23], which is used to compensate for non-homogeneity in the electrochemical system.

The corresponding fitting results are listed in Table 4. *R*_s_ continuously increased with immersion time, it indicated that there was slight change for solution property in different immersion stage [24]. A decrease in pH value represents an increase of ion concentration (Figure 3), which leads to increased conductivity of solution. *R*_t_ value increased first and then decreased, reaching the maximum value on the 21st day. Theoretically, *R*_t_ was only determined by the charge-transfer-controlled corrosion and inversely proportional to *V*_corr_ [25]. Thus, the change of *R*_t_ demonstrated that *V*_corr_ decreased with time for 21 days and then increased after 21 days. It is probably because corrosion products with higher electrochemical activity might participate in cathodic reaction [26], weakening the protection of the corrosion products film, or corrosion products might fall off from the matrix surface.

### 3.7. Polarization Curves

**Figure 7:**
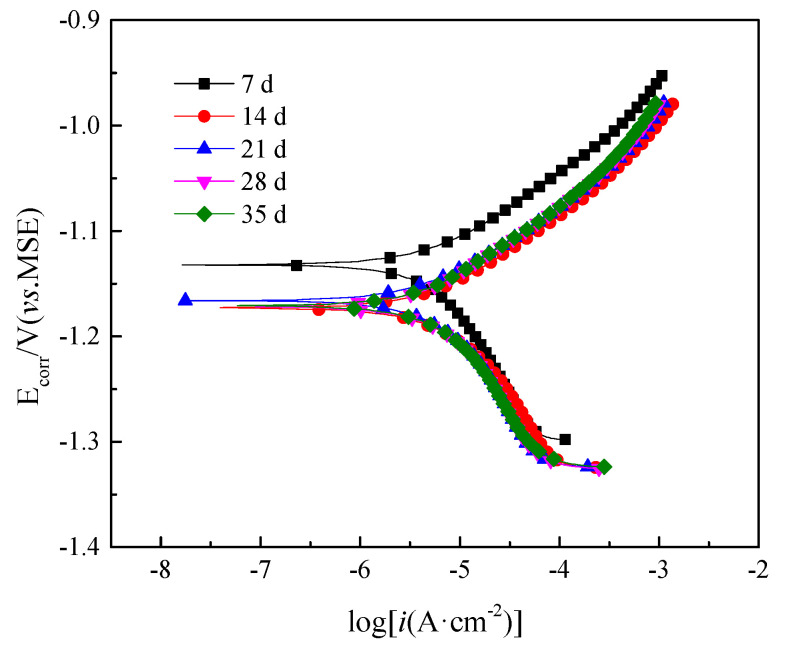
Polarization curves of Q235B steel in desulfurization solution with different immersion time.

Figure 7 showed the polarization curves obtained from Q235B steel in desulfurization solution with different immersion time. As shown in Figure 7, the shape of the anodic and cathodic was similar over the potential domain tested for both samples. In general, a continuous augment in the current density of the cathodic branches was noted as the potential decreased. Because the cathodic branches represented the hydrogen evolution, such as the augment of hydroxide ion [27]. Anodic branches shifted to a great current region, appearing the similar tendency as that observed in cathodic branches. This was due to the cause that the declined pH of the examined solution (Figure 2) caused more severe acidic environments and, with increasing the measurement time, so either transformation of the corrosion products composition (Figure 2) or physical structure of the passive film changed (Figure 1) [28].

The corresponding electrochemical parameters are presented in Table 5, where *E*_corr_ is the corrosion potential, *i*_corr_ (reckoned from intercept of anodic and cathodic Tafel curves) is the corrosion current density, *R*_p_ is the polarization resistance. From this table, the *E*_corr_ decreased rapidly with time in the first 14 days of immersion and then slightly increased from 14 days but subsequently decreased from 21 days to 35 days. The *i*_corr_ was approximately correlated with the *V*_corr_ and had inverse change with the *E*_corr_. The fact illustrated that corrosion products film had no protective effect in the first 14 days. However, it decreased the corrosion rate from 14 days to 21 days. The increase of *i*_corr_ also indicated that the steel matrix was suffered from severe corrosion at time from 21 days to 35 days.

On comparative evaluation, the change law of the corrosion rate obtaining from polarization curves was quite different from that of mass loss. As was shown in Table 3, the *V*_corr_ reduced gradually with 35 days of immersion. This was due to the different principle of measurement. For immersion test, the calculated *V*_corr_ was in fact average value, which should be regarded as uniform corrosion rate. On the basis of NACE Standard RP0775-2005, the *V*_corr_ of immersing 35 days was 0.08 mm·a^−1^, which could be accepted. Nevertheless, the *V*_corr_ presented in electrochemical measurement was a state variable, which was close to pitting corrosion rate. It could cause more serious damage and thus should be given more attention.

### 3.8. The Corrosion Mechanism of Q235B Steel

In oxygenated electrolyte solution, anodes and cathodes would separate [29]. The initial anodic and cathodic reactions of Q235B steel in desulfurization solution are primarily presented as follows:

Fe→Fe^2+^ + 2e^−^ (R1)

O_2_ + 4e^−^ + 2H_2_O→4OH^−^ (R2)

Reactions (R1) and (R2) refer to the anodic and cathodic reaction, respectively. Fe(OH)_2_ was formed in the process of ionic migration according to chemical reaction (R3). Further, Fe(OH)_2_ could be produced from the hydrolysis of Fe^2+^ ions in the anodic region, reaction (R4). Thermodynamic and φ_(MSE)_-pH diagrams predicted that Fe(OH)_2_ was not stable under such conditions. It is easy to be oxidized to Fe(OH)_3_ according to chemical reaction (R5).

Fe^2+^ + 2OH^−^→Fe(OH)_2_↓ (R3)

Fe^2+^ + 2H_2_O→Fe(OH)_2_ + 2H^+^ (R4)

4Fe(OH)_2_ + O_2_ + 2H_2_O→4Fe(OH)_3_ (R5)

Based on metal electrochemical corrosion of thermodynamic principles, the initial corrosion products (Figure 2, 7 days) could spontaneously transform into stabilized phase (Figure 2, 35 days) as shown in reactions (R6) and (R7). In addition, SO_3_^2-^ ions suffered from oxidation into SO_4_^2-^ ions by reaction (R8) increasing the concentration of SO_4_^2-^ ions.

2Fe(OH)_3_→Fe_2_O_3_·H_2_O + 2H_2_O (R6)

2Fe(OH)_3_ + Fe^2+^→Fe_3_O_4_ + 2H_2_O + 2H^+^ (R7)

2SO_3_^2−^ + O_2_→2SO_4_^2−^ (R8)

The concentration of H^+^ ions slowly rise as the reactions (R4) and (R7) progress, reducing the pH down from 6.95 to 5.68 (Figure 3). In general, steel would corrode severely in solutions comprising SO_4_^2−^ ions. The *V*_corr_ and *i*_corr_ slowed down generally in the presence of sediments because Fe_2_O_3_·H_2_O and Fe_3_O_4_ improved the compactness of corrosion product films, which compose a barrier from the corrosive ions toward the matrix surface. Moreover, O_2_ was the main cathode depolarizer in this corrosion environment, therefore limit diffusion of O_2_ naturally turn into rate-control step of cathode reaction [30,31]. It is possible that the decrease of oxygen content in the solution would cause the same effect on *V*_corr_. When oxygen content in the solution declined to a certain extent, it is easy to bring out local O_2_ concentration nonuniform underneath the corrosion products film, causing more localized corrosion (Figure 1c,d). Additionally, iron oxides could be analogous to a kind of cathode depolarizer, which resulted in scattered pit cavity [32]. The growth of corrosion pits can produce Fe^2+^ ions, and the insoluble corrosion products might attract anions [33]. The hydrolysis of Fe^2+^ ions could acidize the local region of the pitting hole and induce the migration of anions, leading to the acceleration of pitting corrosion, reaction (R9) [34,35]. If the ratio of Fe^2+^ ions and SO_4_^2-^ ions exceeded the threshold in the local area, to form insoluble corrosion products FeSO_4_·4H_2_O, reaction (R10) [36]. The corrosion products films might fall off from the steel surface with the increase of sediments, which explained that the decrease in quantity of *R*_p_ and *R*_t_ with time from 28 days of immersion (Table 4 and Table 5).

4Fe^2+^ + 4SO_4_^2−^ + 6H_2_O + O_2_→2 Fe_2_O_3_·H_2_O + 4H_2_SO_4_ (R9)

Fe^2+^ + SO_4_^2−^ + 4H_2_O→FeSO_4_·4H_2_O (R10)

## 4. Conclusions

(1) There was scale sediments attached to the surface of the steel. For 7 days of immersion, the sediments patches were connected and distributed on the matrix surface. The compactness of the sediments increased with time. The initial corrosion products was composed of Fe(OH)_2_, Fe(OH)_3_, and Fe_2_O_3_·H_2_O, the end corrosion products consisted of Fe_2_O_3_·H_2_O, FeSO_4_·4H_2_O, and Fe_3_O_4_.
(2) For immersion test, the *V*_corr_ reduced gradually with 35 days of immersion. However, the results of electrochemical measurement showed that the *V*_corr_ was fluctuant in reality.
(3) In the initial immersion stage (7 days), the primary corrosion type was general corrosion, pitting corrosion was slight and dispersed under the sediments. In the later stage of corrosion (35 days), the cyclic regeneration mechanism of acid, induced by oxidation hydrolysis of FeSO_4_, aggravated the pitting corrosion.
(4) Though the sediments attached to the steel surface could inhibit corrosion, pitting corrosion under the sediments would bring about more serious damage (leak of pipeline and increase of equipment fault rate) thus should be given more attention.

## Figures and Tables

**Table 1:** Chemical element composition of Q235B steel (wt%).

C	Al	Si	P	S	Mn	Fe
0.15	0.184	0.128	0.017	0.013	0.218	bal

**Table 2:** The composition and physicochemical properties of the desulfurization solution.

Content	Chemical Formula	Value
	Na^+^	2785.5
	K^+^	540.7
	Mg^2+^	4.7
	Ca^2+^	26.2
	Zn^2+^	90.2
Ionic concentration (mg L^−1^)	SO_4_^2−^	15,300.3
	SO_3_^2−^	9580.5
	NH_4_^+^	224.6
	NO_3_^−^	3.4
	NO_2_^−^	0.3
Organics concentration (mg L^−1^)	PAHs	158.7
Suspended solids concentration (mg L^−1^)		2519.5
pH		6.95 ± 0.08

**Table 3:** Corrosion rate of Q235B steel in desulfurization solution.

Time/d	7	14	21	28	35
*V*_corr_/(mm/a)	0.22	0.15	0.11	0.10	0.08
Δ*V*_corr_/(mm/a)	—	0.07	0.04	0.01	0.02

**Table 4:** Parameters of equivalent circuits obtained by fitting the experimental results of EIS.

Time/d	*R*_s_/(Ω·cm^2^)	*Q*_dl_/(F·cm^−2^)	*n*	*R*_t_/(Ω·cm^2^)
7	14.03 ± 0.15	(4.48 ± 0.03) × 10^−4^	0.7474 ± 0.008	1092 ± 2.6
14	15.68 ± 0.13	(7.82 ± 0.05) × 10^−4^	0.7588 ± 0.005	1224 ± 3.1
21	16.63 ± 0.11	(9.01 ± 0.07) × 10^−4^	0.7988 ± 0.003	1545 ± 1.8
28	17.77 ± 0.13	(1.29 ± 0.08) × 10^−4^	0.7784 ± 0.005	1469 ± 2.5
35	20.01 ± 0.09	(1.61 ± 0.05) × 10^−4^	0.7619 ± 0.006	1430 ± 2.7

**Table 5:** Parameters of polarization curves with different immersion time.

	7 days	14 days	21 days	28 days	35 days
*E*_corr_ V	−1.133	−1.174	−1.167	−1.172	−1.171
*i*_corr_ μA/cm^2^	7.231	8.074	6.690	7.532	7.459
*R*_p_ Ω·cm^2^	3607	3231	3899	3497	3463
